# *MetaStrainer*: accurate reconstruction of bacterial strain genotypes from short-read metagenomic samples

**DOI:** 10.1093/bioinformatics/btag340

**Published:** 2026-05-24

**Authors:** Hazem Sharaf, Louis-Marie Bobay

**Affiliations:** Department of Biological Sciences, North Carolina State University, Raleigh, NC 27695, United States; Bioinformatic Research Center, North Carolina State University, Raleigh, NC 27695, United States; Department of Biological Sciences, North Carolina State University, Raleigh, NC 27695, United States; Bioinformatic Research Center, North Carolina State University, Raleigh, NC 27695, United States

## Abstract

**Motivation:**

Metagenomics provides broad insights from microbial communities, but more biological relevant phenotypes are attributed to subtle changes at the strain-level rather than species. Despite development of several tools using different algorithms, resolving individual strains from short-read pair-end sequencing data remains challenging.

**Results:**

Here we present *MetaStrainer*, a tool capable of reconstructing strain genotypes from metagenomic data. Compared with existing approaches, *MetaStrainer* substantially increases genotype accuracy, correctly identifies the number of strains, and accurately estimates their relative abundances. Accuracy of reconstructed genotypes is robust to choice of mapping reference.

**Availability:**

*MetaStrainer* is implemented in Python 3. Source code and instructions are available on GitHub at www.github.com/lbobay/MetaStrainer and on Zenodo: 10.5281/zenodo.17872331.

## 1 Introduction

Metagenomic sequencing allows to explore the taxonomic composition, the structure, and the role that entire microbial communities play in their natural environment. Bacterial communities fulfil many key functions across virtually all environments. For example, studies of the human gut microbiome have revealed numerous associations between taxonomic composition and various health conditions (Armour *et al.* 2019). However, despite substantial evidence supporting the role of the microbiome in human health, many studies still fail to identify specific microbial species associated with these conditions. This shortcoming has been attributed, in part, to the vast genomic variability that exists among strains of a single species (Vatanen *et al.* 2019, Yan *et al.* 2020). Indeed, two conspecific strains can differ by >30% of their gene content and display distinct phenotypes, including metabolic functions, antibiotic resistance profiles and virulence factors (Touchon *et al.* 2009). These findings highlight that the characterization of strains—rather than species—is essential for understanding the role that bacteria play in their environments.

Although metagenomic studies have provided many insights regarding microbial communities, analyzing these datasets still constitutes a major challenge. Multiple tools have been developed to assemble short reads de novo and generate Metagenome Assembled Genomes (MAGs) (Li *et al.* 2015, Nurk *et al.* 2017). However, since metagenomic assemblers are largely unable to resolve individual genotypes in the presence of multiple related strains or species, the resulting MAGs capture only consensus sequences and not the underlying strain or even species genotypes. To address this issue, several bioinformatic tools have been developed to characterize strains within metagenomic samples (Anyansi *et al.* 2020). These approaches fall into two categories: (i) tools that identify strains using a set of gene markers and a database of Single Nucleotide Variant (SNV) markers (e.g. *MEGAN*, *StrainPhlAn*) and (ii) tools that aim to reconstruct full strain genotypes using read mapping (Huson *et al.* 2007, Truong *et al.* 2017, Ventolero *et al.* 2022). This latter category is more ambitious, and very few methods exist. Current tools like *StrainFinder* and *StrainFacts* rely on Expectation-Maximization (EM) algorithms to assign alleles to distinct genotypes based on allele frequencies (Smillie *et al.* 2018, Smith *et al.* 2022). However, these tools were originally developed to analyze multiple samples sharing identical strains and are not intended to reconstruct strain genotypes from samples with independent strain compositions. More recently, *mixtureS* (also EM based), was released to reconstruct strain genotypes from independent samples (Li *et al*. 2021). Despite this progress, inferring strain genotypes from short read metagenomic data remains challenging. Consequently, many research teams have increased their efforts to isolate and sequence individual strains, aiming to simplify the inference of strain genotypes from metagenomic data (Poyet *et al.* 2019, Zhao *et al.* 2019, Baker *et al.* 2025). Recently, a novel tool, *PHLAME* (Qu *et al.* 2025), has been developed to identify strains by matching metagenomic samples against a reference database, while also accounting for novel variants. Although these methods offer significant benefits, they rely on establishing extensive genomic datasets, and the process of isolating such a wide range of strains is highly labor-intensive, expensive, and time-consuming.

We developed *MetaStrainer*, a tool that infers genome-wide strain genotypes from short read metagenomic data with high accuracy. *MetaStrainer* uses a framework distinct from previous approaches. First, it leverages paired-end read mapping to generate linkage groups of alleles. Second, it explores a wide range of possible strain distributions using a Markov chain Monte Carlo (MCMC) search to identify the optimal strain composition and optimal strain phasing.

## 2 Methods

The *MetaStrainer* process is summarized Supplementary Fig. S1. *MetaStrainer* requires a user-provided genome as a mapping reference in GenBank format (gbff), and two paired-end metagenomic fastq files (forward and reverse reads). *MetaStrainer* can run with both complete and contig-level assemblies as a reference for read mapping. Because individual strains of bacteria often differ in gene content, the reference genome is first pre-processed to generate a database of individual genes against which the reads will be mapped. Due to the nature of the read-mapping process and tapering coverage towards flanking ends, each reference gene is extended by adding flanking regions equal to the length of sequencing reads on both the 5’ and 3’ ends of each gene (e.g. 150 bp on each side for 150 bp reads, or less if the gene is near the end of a contig). By default, *MetaStrainer* assumes a read length of 150 bp. *MetaStrainer* conducts the mapping of the reads against the reference database using *Bowtie2* with the -very-sensitve -no-unal options by default (Langmead and Salzberg 2012). These options conduct a more extensive search to identify the best alignment and discard unmapped reads from the output file, respectively. *MetaStrainer* interrupts sample processing when less than 60% of the mapping reference is covered as it typically indicates that the mapping reference is too distant to yield accurate genotypes (i.e. a different species). Variant calling is then conducted on the reads, mapping the genes with a minimum base phred quality score of 30 and allele frequency between 1% and 99%. Flanking regions and gene positions with abnormally high or abnormally low coverage are excluded (customizable with a default at 1.5 standard deviations from the mean depth), and a minimum coverage of 20 reads per allele is required. These filters have been implemented to exclude regions with incorrect mapping which could be caused by gene duplications, gene deletions and cross mapping issues from related species. All allele variants detected within the same read or between paired reads are then linked into allele pairs (i.e. allele variants that are known to be physically associated). All the allele pairs are then joined together into linkage groups, which can span multiple genes. Linkage groups are built by first locating the inferred allele variants along each read that successfully mapped a reference gene. All pairs of alleles present within the same read or between pairs reads are then inferred as being part of the same linkage group. This results in linkage groups that could span multiple genes. In addition, non-reference alleles with 100% frequency are also tracked as they represent alleles that are specific to the mapping reference that are not shared with the strains in the sample.

After constructing the linkage groups and computing their frequencies, *MetaStrainer’*s core algorithm is initiated by first assuming that three strains are present in the sample at equal frequencies. The three strains yield a hexamodal distribution where three peaks correspond to the frequencies of the alleles specific to strains 1, 2 and 3, respectively and three additional peaks correspond to the alleles shared by strains 1–2, 1–3 and 2–3, respectively (i.e. corresponding to the frequencies of alleles shared between each pair of strains). *MetaStrainer* then computes the distance *d_k_* of the frequency of each allele pair *k* to the frequency of the closest peak, while doing the same for the complementary alleles. In the case where the complementary alleles are assigned to an incompatible peak (e.g. peak 1 and peak 1–3), the distance *d_k_* is arbitrarily set to 1. Finally, an overall score *S* is computed by summing the distances of all allele pairs *S=∑d_k_*. A MCMC search is then conducted using the MCMC sampler implemented in the Python package *emcee* (Foreman-Mackey *et al.* 2013). The sampler is initialized with 500 random walkers following a Gaussian distribution, and with another sampler run from which random movements are drawn. The main algorithm iterates by drawing movements from these distributions, thereby adjusting the frequency of each strain genotype. After each movement, a slightly different hexamodal distribution of peaks is resampled using the MCMC sampler and the score *S* is recomputed on the new distribution. The mean frequency of two random peaks is shifted using two random values generated using a Gaussian of mean 0.002 and with a standard deviation of 0.002. The value of the third peak is defined as the complement (i.e. the sum of three peaks is equal to 1). The new distribution is accepted if the score *S* is lower than the previous step. Larger jumps are regularly attempted by randomly selecting four types of jumps: every 10 iterations, the resampling of the means of the peaks is randomly increased by a factor 5, 10 or 100 or a new distribution is generated entirely (one of these four possible options is selected at random with equal probability). The same procedure of big jumps is initiated when 20 consecutive iterations have not identified a better distribution (i.e. the score *S* did not improve for 20 consecutive iterations). The algorithm keeps on iterating until it converges towards a stable solution, which is defined by default as a series of 100 consecutive iterations where no better distributions are found (i.e. the score *S* did not improve).

In the final step, *MetaStrainer* reconstructs the genotypes from the strain distribution, the allele frequencies and the linkage groups. Using the allele-assigned hexamodal peaks, *MetaStrainer* classifies the entailed allele to each strain based on a local confidence score which reflects how many times the allele is consistently paired in a relevant peak based on the hexamodal distributions. Alleles that are assigned to a different peak over 50% of the time relative to the other linked alleles in the linkage group are considered ambiguous and thus the allele is set to “N”. In order to avoid overestimating the number of strains due to small genotype differences, the reconstructed genotypes are then compared to one another based on a genome-wide average nucleotide identity. Nearly identical genotypes are collapsed into a single genotype (the most abundant one) using a customizable option with a default set at 99.5% genome-wide nucleotide identity, which has been the recommended threshold for strain definition (Viver *et al.* 2024).

Reads were simulated from *Gilliamella apicola*, a bacterial species that is part of the conserved core bee gut microbiome. The core genome of *G. apicola* was obtained from (Stott *et al.* 2024). The core genome was generated using *CoreCruncher* (Harris *et al.* 2021). Reference strains were selected with an average nucleotide identity above 98% across their core genes (Supplementary Table S2). Six genomes were selected, two of them were used as a mapping reference for aligning the Fastq reads, and the other four to generate simulated populations (Supplementary Table S1). Mapping strain A8 was selected as it had a 99.7% identity to A-1–24 strain, which is above the default strain delineation threshold of 99.5% we use to genotype strains.

Most species in microbiomes are usually dominated by a single strain, but several other less abundant strains may exist at much lower frequencies (Truong *et al.* 2017, Bobay *et al*. 2020). We thus generated samples composed of one to four strains at various relative frequencies (Supplementary Tables S3–5). *CAMISIM* was used to generate the simulated reads. For each simulated population, a configuration file with strain abundances was generated (Supplementary Tables S3–5). The simulation coverage for all strains in the populations is given Supplementary Tables S6–9. Illumina built-in error profile “hi150” was used to simulate 150 bp reads sequenced with a HiSeq. A mean fragment size of 300 bp and standard deviation of 30 bp were used. All other settings were left at default. Prior to analysis, the raw simulated datasets were filtered from low quality reads using *Trimmomatic v0.39* and the following parameters: LEADING: 3 TRAILING: 3 SLIDINGWINDOW: 4:15 MINLEN: 100 AVGQUAL: 30 (Bolger *et al*. 2014).

To evaluate the genotype reconstruction accuracy of each tool, a gene was assessed only if it was present in all simulated reference strains (e.g. present in all three strains of the 3-strain simulations). All gene sequences of the reconstructed strains and reference strains were extracted and then aligned using *muscle v5.1* (Edgar 2022). Reconstructed strains were matched to the original strain using the estimated frequency and the accuracy score. The accuracy score was calculated from all genome-wide variants, i.e. positions with different alleles between the reference strains and the mapping reference used. Positions including alignment gaps “-,” and ambiguous calls “N” were excluded. Any position that did not include a predicted allele for a given tool was assigned the mapping reference corresponding allele and tested as such.

**Figure 1:**
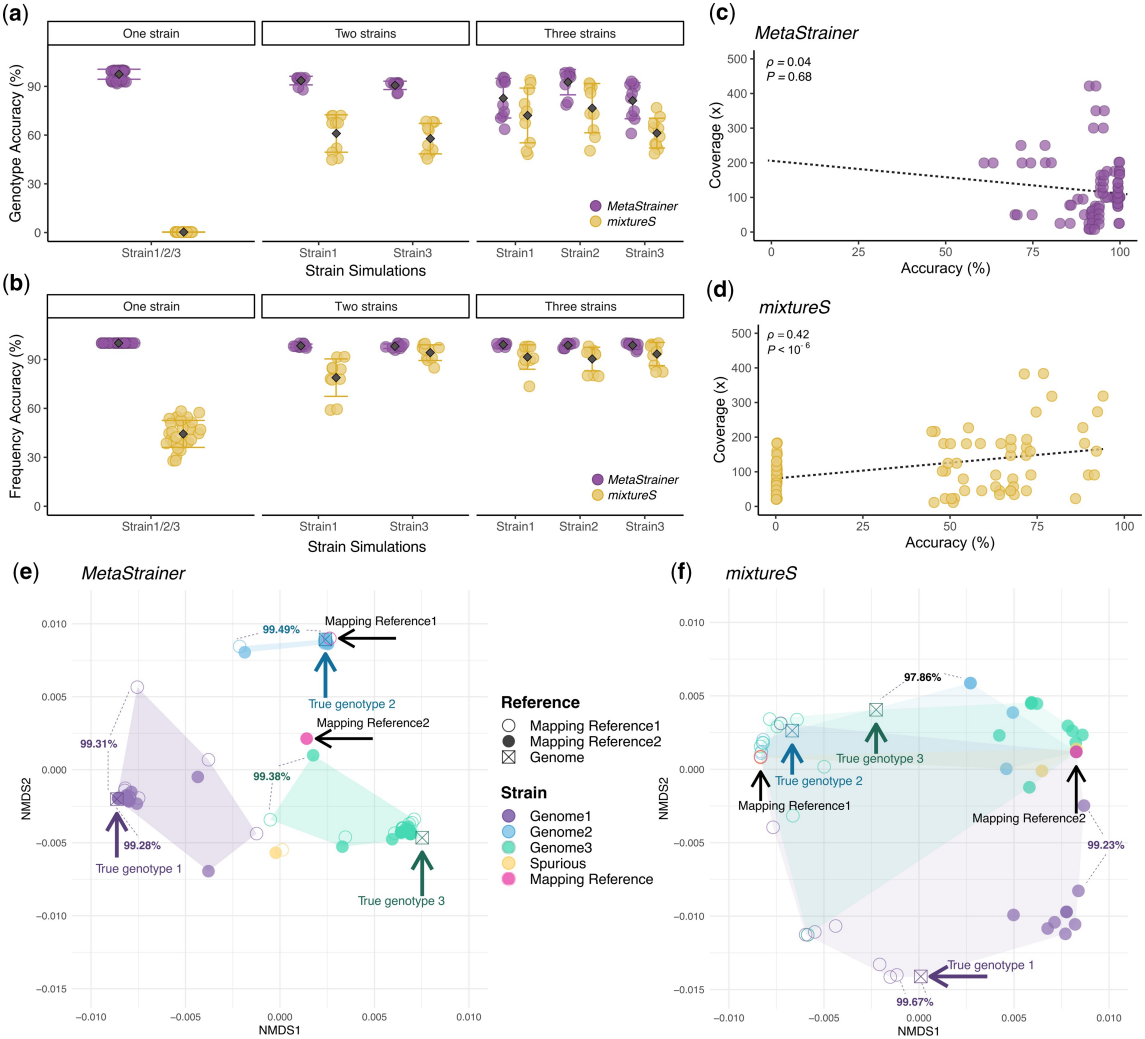
Performance of *MetaStrainer* and *mixtureS*. (a) Accuracy of genotype reconstructions. (b) Accuracy of the prediction of strain frequencies. (c, d) Impact of sequencing coverage on genotyping accuracy for *MetaStrainer* (c) *mixtureS* (d), respectively. (e, f) Non-metric multidimensional scaling (NMDS) ordinations measuring the relatedness of the reconstructed genotypes relative to the mapping references and the true genotypes for MetaStrainer (e) and mixtureS (f), respectively. Average nucleotide identity (ANI) is shown for selected pair of strains.

To estimate the effect of the mapping reference on the tool’s accuracy (Fig. 1e and f), the orthologous genes of the two mapping references and the inferred genotypes were identified with *CoreCruncher*. The genes of all genotypes and references were then concatenated and aligned using *mafft* with the ‘–inputorder’ option (Katoh and Standley 2013). The concatenated alignments for each tool were imported in R using the *Ape* library version 5.6.2 (Paradis *et al*. 2004). A Hamming distance matrix was generated using the dist.dna() function with model=”raw” and pairwise.deletions=TRUE. Nonmetric Multidimensional Scaling (NMDS) was done using the *vegan* package version 2.6.2 metaMDS() function with *k* = 3 (Oksanen *et al.* 2022).

## 3 Results

We tested the performance of *MetaStrainer* using simulated metagenomes and compared its predictions to those obtained with *mixtureS* on the same metagenomes. Multiple metagenomic samples were simulated from full genomes of several strains of the bacteria *G. apicola* (Supplementary Table S1) using *CAMISIM* (Fritz *et al.* 2019). We tested the tools using two reference genomes (strains A8 and N-22, Supplementary Table S1) for read mapping. Each simulated population contained between one and three strains for a total of 56 simulations with various strain frequencies and sequencing depths (Supplementary Tables S3–5). In these simulations, *MetaStrainer* was able to infer the correct number of strains 53 out of 56 times (95%), whereas *mixtureS* predicted the correct number of strains only 4 out of 54 samples it processed (7%). The mean accuracy of strain frequency estimates for *MetaStrainer* was 99.1% and it was 71.3% for *mixtureS* (Fig. 1b). We then evaluated genotype reconstruction accuracy, defined as the percentage of allelic variants correctly inferred. *MetaStrainer* recovered 92.1% of the true genome variants compared to 39.3% for *mixtureS* (Fig. 1a). As opposed to *mixtureS*, *MetaStrainer* does not genotype variants when the signal is too ambiguous (see Methods), and the unpredicted variants are designated as “N”. However, across the genotypes reconstructed with *MetaStrainer*, the number of uncalled alleles varied from only 0 to ∼200 (<0.86% of all variants) across samples.

We tested the impact of sequencing coverage on the ability of *MetaStrainer* to accurately reconstruct genotypes. We found no significant decrease in *MetaStrainer* accuracy relative to the sequencing coverage of each strain (Spearman’s *Rho *= 0.04, *P *= 0.69, Fig. 1c), which was as low as 8× (5% frequency) for some strains. In contrast, *mixtureS*'s accuracy was affected by coverage depth (Spearmans *Rho *= 0.42, *P *< 10^−6^, Fig. 1d, Supplementary Table S7). The robustness of *MetraStrainer* to sequencing coverage is likely due to its reliance on linkage groups rather than individual alleles. Indeed, samples with low coverage display higher variance of allele frequencies, which is partially alleviated by merging alleles into linkage groups.

We further analyzed how the choice of reference genome used for read mapping affected the accuracy of the genotype reconstruction. The two reference genomes used—strain A8 and strain N-22—share 98.8% identity across their core genome (Supplementary Table S6). By design, strain A8 was chosen because it is nearly identical to one of the strains included in the simulated metagenomes. The PCoA plots in Fig. 1d and e give an overview of the relationship of the genotypes reconstructed by *MetaStrainer* and *mixtureS* relative to the two mapping references and the true genomes used to simulate the three strains (i.e. what the reconstructed genotypes should be if the reconstructions were 100% accurate). *MetaStrainer* produced highly similar genotype and frequency estimates regardless of the reference genome used (*R *= 0, *P *= .4, ANOSIM, Fig. 1d). In contrast, the genotypes reconstructed by *mixtureS* were strongly affected by the choice of the reference used for the mapping (*R *= 0.94, *P *= .001, ANOSIM, Fig. 1e). Overall, *MetaStrainer* is highly robust to the choice of the reference genome used for the mapping.

Natural microbial communities are typically dominated by a single or a few strains per species, although additional strains can be present at very low frequencies (Truong *et al.* 2017, Bobay *et al*. 2020). The current implementation of *MetaStrainer* is limited to inferring a maximum of three strains. Alternative tools—such as *mixtureS*—do not have this limitation and are therefore expected to outperform *MetaStrainer* when samples are composed of four strains or more. To evaluate this, we tested both tools on simulated metagenomes composed of four strains (Supplementary Tables S1–5 and Fig. S2). *MetaStrainer* detected three strains (its maximum) in nine out of ten simulations, with a mean accuracy of 81.7%, whereas *mixtureS* detected at least four strains six times out of ten with a mean accuracy of 69.0%. *MetaStrainer* achieved more than 90% genotype accuracy for the dominant strain in six simulations (Supplementary Fig. S2c). Therefore, although *MetaStrainer* does not attempt to reconstruct all the strains present in the sample in these situations, those genotypes that are reconstructed are highly accurate. Note that, as a default parameter, strains that share ≥99.5% overall identity are merged into a single strain. Across our simulations composed of a single strain or two strains, 46 strains were merged because they were virtually identical to another strain (99.93% on average, min = 99.51%, max = 99.99%). Although it is possible for users to attempt to define strains at a finer resolution, users should only do so on samples with very high quality and very low complexity.

Finally, because all strain-reconstruction algorithms rely on allele frequencies, their accuracy is expected to decline when strains are present at similar frequencies in a sample. We therefore tested *MetaStrainer* on the most challenging scenarios: strains present at equal frequencies (e.g. 1/3, 1/3, 1/3). As expected, the overall accuracy of the inferred genotypes was much lower (<70% of variants called accurately) in these extreme cases. Nevertheless, *MetaStrainer* still outperformed *mixtureS* in this situation (64.2% and 53.4% predicted variants, respectively, Supplementary Fig. S3).

Reconstructing the genotypes of larger numbers of strains remains challenging for *MetaStrainer* as well as for other approaches, including phasing or assembly-based methods, even those using long reads (Garud *et al.* 2019, Shaw *et al.* 2024). *MetaStrainer* and other algorithms are less accurate at reconstructing strain genotypes when (i) many strains are present in a sample (≥4 strains) and (ii) two or more strains occur at similar frequencies in a sample. Although it is theoretically possible to expand our implementation to infer additional strains, our preliminary attempts revealed that it added a substantial cost to the accuracy and computational time of our approach. In natural communities, most bacterial species are dominated by a single or very few strains and instances where four strains are present in a sample and where each strain composes over 5% of the population are largely unexpected to occur (Truong *et al.* 2017, Bobay *et al*. 2020). This suggests that methods focusing on a smaller—but more accurate—number of reconstructed strains are most relevant. Newer tools have been developed using different approaches, such as phasing by minimum error correction of allele counts, and can more accurately identify the number of strains (Shaw *et al.* 2024). Therefore, we recommend using these tools prior to running *MetaStrainer* to estimate the number of expected strains in a sample. It should be strongly emphasized that, since *MetaStrainer* is a mapping-based approach, the species composition of the sample(s) should be accurately characterized prior to running *MetaStrainer*. The presence of closely related species in a sample can potentially lead to incorrect mapping, and, in such cases, we strongly recommend conducting the analyses with a reference genome of each related species to prevent cross-mapping issues. Note that *MetaStrainer* is agnostic to the concept of species and it may resolve the genotypes of species rather than strains when closely related species are present. *MetaStrainer* is defining strains with a default threshold of 99.5% ANI, which has been recently recommended as a “natural” threshold for strain definition (Viver *et al.* 2024). Users should adjust this threshold according to the quality and the complexity of the samples (i.e. samples with high coverage and few strains could be used to resolve strain genotypes with fewer variants). Finally, it should be noted that, because strains differ in gene content, mapping-based approaches like *MetaStrainer* are not able to reconstruct the genes absent in a reference genome. Users should therefore expect to primarily reconstruct the core genome of the strains and closely related strains that differ in gene content but that have nearly identical core genomes will likely be merged into a single genotype by *MetaStrainer*. However, this last issue is unlikely to impact the inference of *MetaStrainer* since strains with nearly identical core genomes also share nearly identical gene contents (Croucher *et al.* 2014). Overall, *MetaStrainer* is capable of reconstructing bacterial strain genotypes from metagenomic reads with high accuracy in most situations and outperforms the current tools for strain genotype reconstruction.

## Supplementary Material

btag340_Supplementary_Data

## Data Availability

All the scripts and instructions are freely available on GitHub at www.github.com/lbobay/MetaStrainer and on Zenodo: 10.5281/zenodo.17872331.
