# Supplementary material for: MetaStrainer: accurate reconstruction of bacterial strain genotypes from short-read metagenomic samples
Source: Bioinformatics. 2026 May 24;42(6):btag340. doi: 10.1093/bioinformatics/btag340 (PMC13224963; doi:10.1093/bioinformatics/btag340)
Supplement: btag340_Supplementary_Data [file btag340_supplementary_data.pdf]

Supplementary Materials for

***MetaStrainer*: Accurate reconstruction of bacterial strain genotypes from short-read metagenomic samples.**

Hazem Sharaf[1,2] and Louis-Marie Bobay[1,2,*]

[1]Department of Biological Sciences, North Carolina State University, Raleigh, United States

[2]Bioinformatic Research Center, North Carolina State University, Raleigh, United States

[*] Corresponding author: Louis-Marie Bobay

Contact details:

1 Lampe Dr.

27697, Raleigh, NC

ljbobay@ncsu.edu

1. Input FASTQ files and reference genome

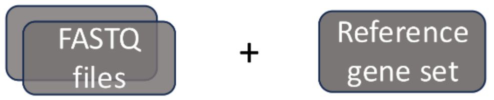

2. Add flanking regions and create mapping reference

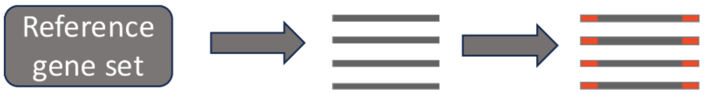

3. Map to reference gene set with extra flank regions

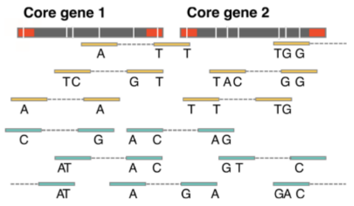

4. Infer variants

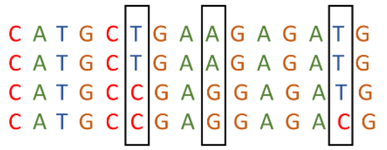

5. Generate linkage groups

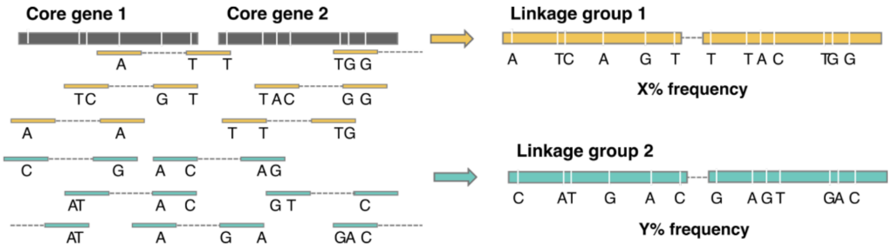

6. Run MCMC search

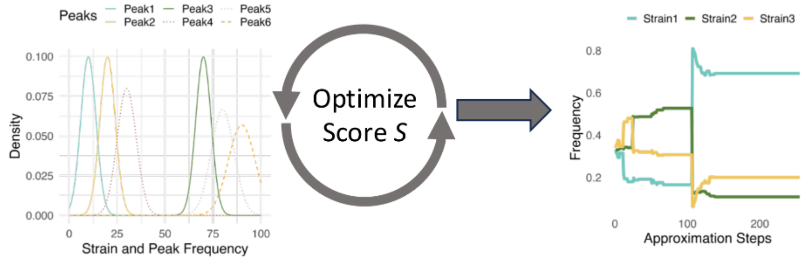

7. Generate strain genotypes

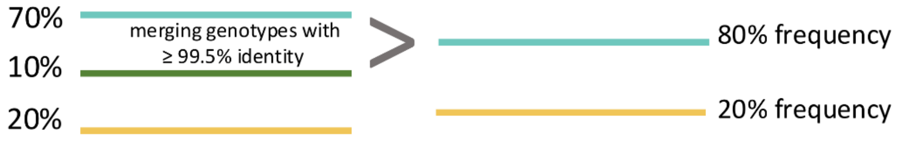

Figure S1: A schematic overview summarizing the workflow of *MetaStrainer*. The first step starts with a user provided quality-controlled FASTQ file and a reference genome. In the second step genes are extracted and filtered, then flanking regions (150 bp) are added to each gene to construct an expanded mapping reference. In the third step, reads are mapped to that reference. In the fourth step, variants within individual and paired reads are linked together. In the fifth step, these links are used to build linkage groups from genes spanned by read-pairs and allele-pairs. In the sixth step, *MetaStrainer* explore diverse hexamodal distributions corresponding to the genotype frequencies of three strains with a MCMC search. For each distribution, alleles from a linkage group are assigned to their closest peak and the score *S* is computed as the sum of distances of the frequencies of each allele to their closest peak. Different distributions are explored until the score *S* is stable (100 iterations without identifying a better score). Finally, in the seventh step, linkage groups are broken down and strains are reconstructed from peak-allele assignments. Strains with >99.5% (default) similarity are merged together.

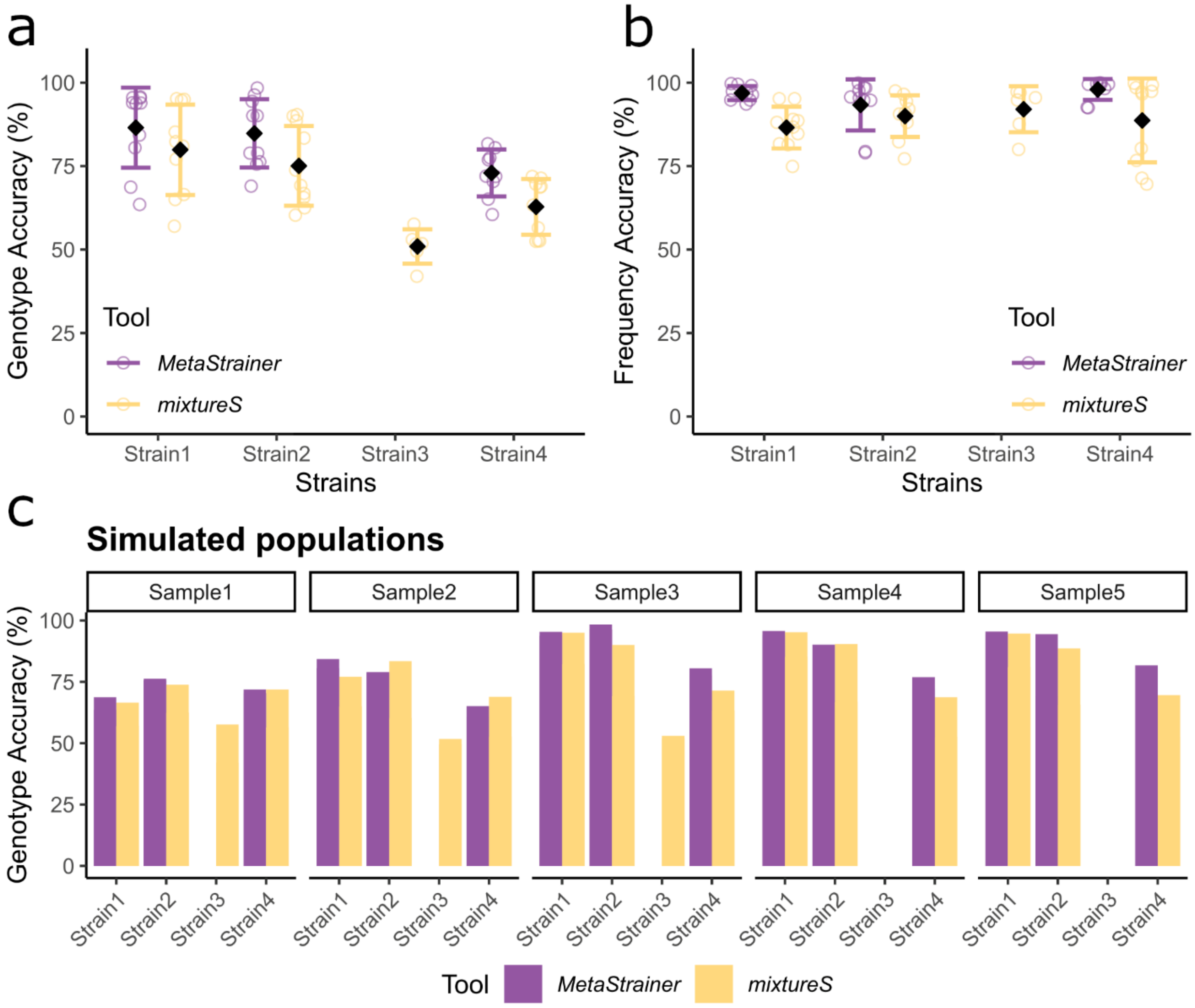

Figure S2: Genotype accuracy (a) and frequency accuracy (b) of *MetaStrainer* and *mixtureS* for samples simulated with four strains. Genotype accuracy (c) for each the populations simulated with four strains.

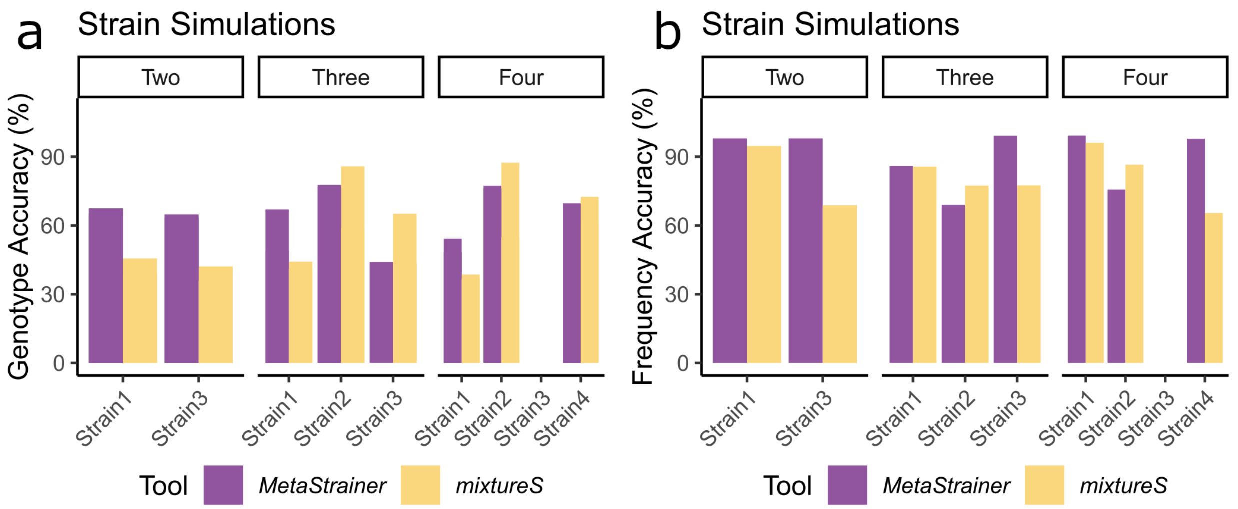

Figure S3: Genotype accuracy (a) and frequency accuracy (b) of *MetaStrainer* and *mixtureS* on samples simulated with strains at equal frequencies.

Table S1: *Gilliamella apicola* strains used in this study

Genome	Strain	Purpose
GCA_001690735.1	P54G	Simulation Reference
GCA_002141515.1	A-1-24	Simulation Reference
GCA_000599985.1	wkB1*	Simulation Reference
GCA_003202815.1	ESL0178	Simulation Reference
GCA_002141555.1	A8	Alignment Reference
GCA_002142285.1	N-22	Alignment Reference

* Typer material strain assembly

Table S2: Pairwise nucleotide identity between the core-genomes of *G. apicola* strains used in this study

	GCA_002141515.1	GCA_000599985.1	GCA_003202815.1	GCA_002141555.1	GCA_002142285.1
GCA_001690735.1	98.30%	98.00%	98.30%	98.30%	98.20%
GCA_002141515.1		98.50%	98.80%	99.70%	98.70%
GCA_000599985.1			98.40%	98.50%	98.20%
GCA_003202815.1				98.70%	98.50%
GCA_002141555.1					98.60%

Table S3: 3-strain simulations with the frequency of each *G. apicola* strain in each sample

Genome	Sample1	Sample2	Sample3	Sample4	Sample5	Sample6
GCA_001690735.1	0.4	0.7	0.65	0.5	0.34	0.1
GCA_002141515.1	0.2	0.2	0.3	0.4	0.33	0.05
GCA_000599985.1	0.4	0.1	0.05	0.1	0.33	0.85

Table S4: 2-strain simulations with the frequency of each *G. apicola* strain in each sample

Genome	Sample1	Sample2	Sample3	Sample4	Sample5	Sample6
GCA_001690735.1	0.5	0.55	0.65	0.75	0.85	0.95
GCA_000599985.1	0.5	0.45	0.35	0.25	0.15	0.05

Table S5: 4-strain simulations with the frequency of each *G. apicola* strain in each sample

Genome	Sample1	Sample2	Sample3	Sample4	Sample5	Sample6
GCA_001690735.1	0.4	0.5	0.6	0.7	0.8	0.25
GCA_002141515.1	0.3	0.25	0.25	0.15	0.12	0.25
GCA_000599985.1	0.1	0.1	0.05	0.05	0.02	0.25
GCA_003202815.1	0.2	0.15	0.1	0.1	0.06	0.25

Table S6: The simulated sequencing coverage generated using CAMISIM of the genomes used to generate the populations of 3-strain simulations

Genome	Sample1	Sample2	Sample3	Sample4	Sample5	Sample6
GCA_001690735.1	255.8	449.2	384.5	320.1	217.3	63.8
GCA_002141515.1	127.9	128.3	224.3	256.0	210.9	31.9
GCA_000599985.1	255.8	64.2	32.0	64.0	210.9	542.0

Table S7: The simulated sequencing coverage generated using CAMISIM of the genomes used to generate the populations of 2-strain simulations

Genome	Sample1	Sample2	Sample3	Sample4	Sample5	Sample6
GCA_001690735.1	162.1	178.2	210.3	242.3	274.3	306.1
GCA_000599985.1	162.1	145.8	113.2	80.8	48.4	16.1

Table S8: The simulated sequencing coverage generated using CAMISIM of the genomes used to generate the populations of 4-strain simulations

Genome	Sample1	Sample2	Sample3	Sample4	Sample5	Sample6
GCA_001690735.1	391.0	487.3	582.3	680.2	775.2	245.9
GCA_002141515.1	293.2	243.7	242.6	145.8	116.3	245.9
GCA_000599985.1	97.7	97.5	48.5	48.6	19.4	245.9
GCA_003202815.1	195.5	146.2	97.1	97.2	58.1	245.9

Table S9: The simulated sequencing coverage generated using CAMISIM of individual genomes used in single strain populations simulations

Genome	Sample1	Sample2	Sample3	Sample4	Sample5	Sample6
GCA_001690735.1	96.1	105.7	125.1	144.5	164.0	183.4
GCA_002141515.1	127.9	128.3	224.3	256.0	210.9	31.9
GCA_000599985.1	96.1	86.5	67.4	48.2	28.9	9.7
